# A qualitative exploration of mental health workers trained and supported to deliver a cognitive remediation program, Employ Your Mind (EYM)

**DOI:** 10.3389/fpsyt.2026.1891467

**Published:** 2026-09-14

**Authors:** Kayla Ho, Kirsty Pope, Anne Miles

**Affiliations:** 1 Department of Occupational Therapy, Monash University, Frankston, VIC, Australia; 2 WISE IDEA, WISE, Melbourne, VIC, Australia

**Keywords:** cognitive remediation, implementation, Kirkpatrick model of evaluation, mental health condition, severe mental illness, training, vocational rehabilitation

## Abstract

**Background:**

Cognitive remediation is an evidence-based intervention designed to improve the functional cognition of people with mental health conditions. Employ Your Mind is a vocational rehabilitation program with embedded cognitive remediation that is facilitated by trained mental health workers, referred to as Learning Coaches within this context. Providing effective training and support to Learning Coaches forms a significant component to the implementation of the program. To date, there has been limited examination of Learning Coaches’ experiences of delivering the Employ Your Mind program. These perspectives are important for the wider implementation of cognitive remediation interventions to improve access for people with mental health conditions.

**Methods:**

Semi-structured interviews were conducted between March–April (2025) with purposively sampled Employ Your Mind Learning Coaches and their managers, from three community-based mental health organizations involved in a 12-month Employ Your Mind Demonstration Project (2023-2024) led by WISE IDEA. Qualitative data underwent reflexive thematic analysis procedures and were compared against the Kirkpatrick Model of Evaluation.

**Results:**

Learning Coaches and their managers (N = 9) described their experiences of the Employ Your Mind training and implementation as a journey of learning and skill development. Learning Coaches noted enhancements in their therapeutic approach (Kirkpatrick Model Level 3: Behaviour), improved job satisfaction in observing positive consumer outcomes (Kirkpatrick Model Level 4: Results) and the significance of ongoing support and supervision to navigate implementation challenges.

**Discussion:**

This study informs the development of future training for vocational rehabilitation programs with embedded cognitive remediation. Several enablers for the successful training and implementation of the Employ Your Mind program were identified and are discussed from the Learning Coach and managerial perspective.

## 1 Introduction

People living with mental health conditions experience the lowest rates of employment when compared to those with other disability (1), with employment rates for people diagnosed with schizophrenia being as low as 13% in Australia (2). This is of particular concern, considering the relationship between mental health and employment appears to contribute to better recovery outcomes, and greater quality of life for people with mental health conditions, such as schizophrenia and bipolar disorder (3, 4). Yet commonly, many people with mental health conditions experience reduced cognitive functioning (5) which can affect functional, psychosocial, and vocational outcomes (4–7).

For people with mental health conditions, cognitive processes and vocational outcomes can be improved through interventions such as cognitive remediation (CR). CR targets cognition (e.g., attention, memory, executive function) by utilizing scientific principles of learning to improve functional outcomes and generalize cognitive processes to everyday life (7–9). The efficacy of CR is enhanced when delivered in supportive environments, alongside other forms of psychosocial rehabilitation, including vocational rehabilitation (7, 10). Despite the evidence for CR (11), and recommendations as a treatment for cognitive challenges for people with mental health conditions (12, 13), the availability of CR in Australia remains limited (14). Workforce training and supervision have been identified as key enablers to successful implementation of CR programs both in Australia and internationally (15).

Facilitators of CR serve an active role in supporting people with mental health conditions (referred to as consumers hereafter) to best address their cognitive challenges and promote functional improvements (7, 16). Common CR facilitator training methods involving didactics, role-playing and modules (17–20) appear to enable facilitators to acquire the necessary knowledge and skills to support consumer application of CR into their lives (21, 22). Such training methods reflect experiential learning concepts to ensure facilitators respond effectively to the evolving needs of their consumers (23). Furthermore, ongoing support and supervision are also noted as a necessary component to ongoing CR facilitation to ensure facilitators can maintain program fidelity, in addition to personalizing CR tasks/strategies for optimal delivery and consumer outcomes (19, 24). Beyond facilitators’ engagement in CR training, an additional benefit reported by CR facilitators include experiences of improved job satisfaction throughout delivery (25), highlighting its novelty as an intervention form.

It is evident that facilitators form a significant component of successful CR implementation that supports consumers to achieve psychosocial and vocational goals (21, 26). To date, however, studies investigating CR often focus on outcomes for individuals with severe or complex mental health conditions (6, 7, 9) which provide less emphasis and insight into the feasibility and acceptability (27) of CR training and delivery from facilitator perspectives. Therefore, the optimal training and support methods required to enable facilitators to effectively implement CR remain unclear. This gap in CR literature can be addressed by exploring the experiences and perspectives of CR facilitators.

Within Australia, Employ Your Mind (EYM) is one of the few available vocational rehabilitation programs with embedded CR. Originally developed by Fife Employment Access Trust (FEAT) in Scotland, EYM is delivered in four six-week phases over six-months (see **Table 1**), comprising of individual and group-based activities and computer-based CR exercises (28).

**Table 1:** Outline of EYM program phases and components.

Phase	Delivery and duration	Components
Phase 1 – Explore Your Mind	Individual: one 90-minute session per week for 6 weeks	• Introduction to cognition, CR and EYM program • Completion of cognitive and psychosocial exploration (assessments) • Completion of strength-based character survey • Interest exploration and goal setting
Phase 2 – Share Your Mind	Group: two 90-minute sessions per week for 6 weeks	• Exploration of communication, social cognition, and goal setting in context of work/vocation • Commencement of individual project work • Thinking Gym – on-line CR delivery using HappyNeuron Pro (including strategy coaching and bridging discussions) • End of Phase group celebration and certificate
Phase 3 – Educate Your Mind	Group: two 90-minute sessions per week for 6 weeks	• Exploration of communication, social cognition, problem solving, physical health, cognitive health and goal setting in context of work/vocation • Completion of individual project work and presentation • Thinking Gym – on-line CR delivery using HappyNeuron Pro (including strategy coaching and bridging discussions) • End of Phase group celebration and certificate
Phase 4 – Know Your Mind	Group and Individual: two 90-minute sessions per week for 6 weeks	• Exploration of work orientation topics including motivation for work, job matching, rights & responsibilities, disclosure, managing benefits, and maintaining wellness at work • Thinking Gym – on-line CR delivery using HappyNeuron Pro (including strategy coaching and bridging discussions) • Completion of cognitive and psychosocial exploration including progress discussion and report • Graduation group celebration and certificate

EYM was first piloted in Australia in 2016 (29), leading to the delivery of EYM from 2018–2022 by WISE’s WISE Ways to Work program (former title of WISE IDEA) (28). WISE IDEA is a center of best practice in mental health and employment and specializes in the development, evaluation and implementation of evidence-based vocational programs that mitigate barriers to employment for individuals with severe or complex mental health conditions.

The EYM program is facilitated by Learning Coaches (LCs) who actively guide the program and enable consumers (referred to as Students in the context of EYM) to improve their cognitive and work-related skills and generalize EYM learnings for everyday life and employment (25, 29). EYM has displayed statistically significant improvements in areas such as subjective and objective cognition, quality of life, perception of working ability and mental wellbeing (28, 29).

Despite the recommendations for the provision of CR as an evidence-based intervention for addressing cognitive challenges (12, 13), the implementation of CR in Australia remains limited, restricting access for people who could benefit (14). WISE IDEA developed a ‘Train-the-Coach’ model for EYM for the purpose of building the skills of staff in community and clinical mental health services, to deliver the EYM program and improve the availability of CR interventions.

The current study was an extension of a 12-month EYM Demonstration Project delivered by WISE IDEA between 2023-2024. The Demonstration Project aimed to assess the feasibility, acceptability and implementation of the EYM ‘Train-the-Coach’ model within community based mental health organizations. As part of the Demonstration Project, WISE IDEA provided training, materials and support, including fortnightly group supervision for staff, from three partner community mental health organizations to enable the delivery of EYM in their services.

The ability to facilitate, manage and effectively coach productive EYM sessions requires several skills and abilities. To assist partner organizations to identify and nominate suitable staff to be trained, and for trained staff to have the capability to deliver EYM to the standard expected, WISE IDEA developed an EYM Facilitator Competency Framework and an EYM Fidelity Scale. The EYM Facilitator Competency Framework combines recommendations provided by CR researchers and clinicians with the social/emotional intelligence framework (30). Senior leaders in partner organizations used the WISE IDEA Facilitator Competency Framework, to assist in the selection and invitation of suitable staff to participate in the Demonstration Project.

In accordance with the WISE IDEA Facilitator Competency Framework, LCs are eligible to facilitate EYM independently (known as Facilitator 1) if they hold at least a bachelor’s degree in a mental health field (e.g. occupational therapy, psychology, social work, nursing), in addition to experience in adult mental health group facilitation. LCs are eligible to co-facilitate EYM alongside Facilitator 1s if they have experience in adult mental health group facilitation, but do not hold a bachelor qualification in a mental health field. This co-facilitator role (Facilitator 2) is highly valued and enables other mental health workers, including Lived Experience or Peer Workers to be involved in the facilitation of EYM groups.

The EYM Fidelity Scale was designed to ensure consistent delivery of information, guidance and materials provided by the LCs. It is completed as a self-assessment and incorporates recommendations of the expert working group white paper on core techniques in CR (31). The Fidelity Scale also assists partner organizations to identify strengths and limitations of their EYM delivery and develop plans for improvement.

Nine community-based mental health workers across three organizations were trained as EYM LCs. All LCs completed the initial in-person two-day EYM training, with ongoing half day hybrid trainings (delivered both in person and on-line depending on the LCs’ location) provided prior to the LCs’ delivery of EYM Phases 2-4. All EYM training was delivered by the senior occupational therapist at WISE IDEA (EYM trainer). Day one of the two-day EYM training for LCs included learning the importance of vocation for people with mental health conditions, understanding cognition, and the evidence base and theoretical underpinnings of CR. Day two involved familiarizing LCs with pre-intervention assessments to be administered with EYM Students and the EYM Phase 1 curriculum. The half-day trainings were delivered to LCs ahead of the commencement of each new EYM Phase by the EYM trainer, to ensure they developed a sound understanding of the content and associated materials, as near to the delivery of the next Phase. This intended to promote consolidation of the training for each EYM Phase and ensure effective delivery to EYM Students. Within the half day training sessions, phase-specific overviews of the content, discussion topics, CR coaching methods and support was provided to LCs.

Throughout the facilitation and implementation of EYM by trained LCs, the EYM trainer provided ongoing fortnightly group support and supervision, individual support when required, in addition to interpretation of the EYM assessment results and report writing, required for Phases 1 and 4 of the EYM program. The group supervision component provided ongoing support to LCs once implementation of the EYM program commenced. Using the Proctor’s Model of Supervision (32), delivered through a supportive mentoring and coaching style approach, group supervision sessions were designed to support LCs with competency development, reflective practice, and fidelity to the EYM program and core components of CR. These components include enhancing therapist engagement and competency, effective application of cognitive exercises, and supporting the development and transferability of cognitive strategies to daily functioning (31).

At the completion of EYM program delivery, the LCs completed the EYM Fidelity Scale and engaged in discussions with the EYM trainer about areas of strength and areas for development regarding program facilitation.

This study aimed to examine the experiences of the mental health workers trained and supported to deliver EYM the consumers they work with.

The research questions were:

1. What are the mental health workers’ perceptions of the EYM training?
2. What are the mental health workers’ perceptions of how delivering EYM has changed how they work with consumers?
3. What are managers’ perceptions of service delivery following the EYM training of their mental health workers?

## 2 Materials and methods

This study used a qualitative research design guided by a constructivist-interpretive paradigm. This paradigm is a philosophical research approach that intends to understand human experiences, behaviors and interactions, by emphasizing subjective interpretations, meaning construction and the socio-cultural contexts of individuals to facilitate knowledge development (33). This was appropriate in enabling LCs and their managers to effectively convey, define and interpret their subjective realities and experiences of the EYM training and delivery, alongside the active involvement of the first author and to co-construct findings (34).

Ethics approval was received from the Monash University Human Research Ethics Committee (Project ID: 45014) before the commencement of this study.

### 2.1 Recruitment and sampling

LCs and their managers were recruited from three community-based mental health organizations involved in the 12-month EYM Demonstration Project. Using the WISE IDEA internal communication networks, the third author within the research team sent recruitment emails including the explanatory statement to eligible LCs and managers. LCs and their managers were then required to respond directly to the first author to participate in the study, where written informed consent was obtained from all LCs and their managers. Verbal consent was also obtained from all LCs and managers, prior to semi-structured interviews.

A purposive sampling strategy was used to select participants trained as EYM LCs or as managers of EYM LCs. This sampling strategy was appropriate due to the limited pool of mental health workers and managers involved in the EYM training and delivery, throughout the Demonstration Project (2023–2024) conducted by WISE IDEA. More specifically, the total pool of participants involved in the EYM training and delivery included 13 individuals; nine LCs and three managers.

LCs and their managers were eligible if they met the inclusion criteria (see **Table 2**), and the respective exclusion criteria. Recruiting managers of EYM LCs sought to further understand the LCs’ individual experiences and examine the organizational and implementation challenges of EYM from a managerial perspective.

**Table 2:** Inclusion/exclusion criteria for LCs and managers.

Learning coaches	
Inclusion criteria	Exclusion criteria
Learning Coaches have fully completed the initial EYM two-day training, provided by WISE IDEA.	Mental health workers trained in CR programs other than EYM.
	Only completed Day 1 of the EYM two-day training course.
Managers	
Inclusion Criteria	Exclusion Criteria
Direct involvement in supervising or communicating with their staff who completed the EYM two-day training.	Managers who did not have direct involvement in supervision or communication with their staff, of whom were trained in EYM and deliver the program within their organization.
Direct involvement in the management and delivery of EYM within their organization, since the commencement of the demonstration project in August 2023.	

### 2.2 Data collection

LCs and their managers completed a demographic questionnaire (see **Supplementary Material 1**) and proceeded to engage in a semi-structured interview with the first author between March–April (2025), via videoconferencing or in-person, in accordance with participant preferences. The interview guide (see **Supplementary Material 2**) was designed to reflect the levels within the Kirkpatrick Model of Evaluation (35) (see **Table 3**) and included open-ended and neutrally phrased questions, to encourage participants to report their experiences openly and in alignment with this model. The interview guide aimed to identify the LC and managerial perceptions of the level of change, achieved as a result of the EYM training and delivery. Pilot testing was conducted within the research team to optimize the interview guide prior to formal data collection procedures. Prompts were used throughout participant interviews to clarify or promote further elaboration of participant statements.

**Table 3:** The four levels of the Kirkpatrick model.

Kirkpatrick Level of Evaluation	Evaluation Description for the Current Study
**Level 1: Reaction**	The degree to which Learning Coaches find the training favorable, engaging and relevant to their jobs.
**Level 2: Learning**	The degree to which Learning Coaches acquire the intended knowledge, skills, attitude, confidence and commitment based on their participation in the training.
**Level 3: Behaviour**	The degree to which Learning Coaches apply their learnings from the training when they are back on the job.
**Level 4: Results**	The degree to which targeted outcomes occur as a result of the training experience and the performance support provided by organizational members.

Interviews spanned 11–56 minutes in length, with an average of 38 minutes. To maintain confidentiality, LCs and managers were asked to select a pseudonym, or alternatively, a pseudonym was assigned to the participant.

### 2.3 Data analysis

All interviews were audio-recorded, transcribed verbatim and securely stored on a password-protected Google Drive, accessible only via the research team. Transcripts were reviewed, de-identified and returned to LCs and managers for member-checking, with no further member-checking processes being completed from this point in the study. One participant requested minor amendments to their transcript, to more accurately reflect their experiences. All other participants confirmed the accuracy of the transcripts with no amendments suggested. Transcripts were imported into NVivo 15 software (36) which was used to code but not analyze transcripts. The Kirkpatrick Model of Evaluation was selected to support data analysis procedures, as it provides an adaptable and reliable method of evaluating the effectiveness of a training program (37). Evaluating the EYM training against the Kirkpatrick Model’s levels of Evaluation enabled the research team to examine the impact and level of change experienced by the LCs (38).

Data collection and analysis occurred simultaneously, with reflexive thematic analysis procedures guided by Braun and Clarke (39) completed inductively by the first author. Confirmation bias was minimized through the first author not having been directly involved in the training of EYM LCs or the delivery of the EYM Demonstration Project. Researcher reflexivity was addressed through the first author developing written reflections and field notes following participant interviews and data analysis stages. The research team engaged in frequent peer debriefing to maintain ongoing data familiarization and aid systematic coding. Codes were generated iteratively into initial themes and frequently reviewed to support data accuracy and researcher interpretations (40).

The research team continually refined, defined, and named themes and subheadings to align with the study aim and research questions, to ensure data relevance and closeness (39). The research team agreed on the finalized theme and identified participant quotes to support study findings.

### 2.4 Positionality

The first author (KH) is an early in career occupational therapist who gained research experience throughout her undergraduate degree. KH entered the study with limited pre-existing knowledge of CR interventions or EYM, which meant she had few prior assumptions regarding EYM. To understand the organizational context and the EYM program, the first author (KH) engaged with WISE IDEA on-site over a 12-month period to further understand the nature, purpose and methods of the EYM training and delivery provided to LCs, during the demonstration project. This prolonged engagement was addressed through reflexive journaling and regular team discussions of KH’s observations and its influence on researcher interpretations.

The second author (KP) is an occupational therapy educator and academic who holds experience in mental health and designing, conducting, interpreting and supervising research projects. KP was not involved in the EYM demonstration project but brought experience of using the Kirkpatrick Model within research contexts, which informed the study’s methodology. The third author (AM) is the senior occupational therapist at WISE IDEA who led the provision of the EYM training and ongoing supervision to LCs during their delivery of the intervention. Given this, the research team recognized the potential for social desirability and positive response bias from participants and ensured they were not interviewed by the third author (AM), but rather the first author (KH). The research team engaged in reflexive discussion during data collection and analysis, which allowed for individual interpretations and assumptions to be challenged, and enhance the credibility of study findings.

## 3 Results

In total, nine interviews were conducted with seven LCs and two managers. LCs and managers were mostly women (*n* = 6) and held varying professional roles in their organizations, including recovery specialist (*n =* 2), community mental health practitioner (*n =* 2) and peer leader (*n* = 1) roles. Two LCs did not deliver any component of the EYM program, while five delivered a complete EYM program. Differentiating LCs according to their training and delivery status within the results was deemed unnecessary by the research team, as data collection for LCs who did not deliver the EYM program aimed to identify and explore the organizational factors which prevented EYM implementation, in addition to their experiences of the training. Further demographic data can be found in **Table 4**. Analysis of the interview transcripts generated an overarching theme of the EYM training and implementation as a journey of learning.

**Table 4:** Learning coach and managerial demographic data.

Characteristics	Number of participants (N = 9)
Gender	
Woman	6
Man	3
Age (years)	
30-39	3
40-49	4
50-59	1
60-69	1
Highest level of educational attainment	
Postgraduate Degree	2
Graduate Diploma / Graduate Certificate	1
Bachelor’s Degree	4
Advanced Diploma	2
Discipline of qualification	
Psychology	4
Social Science	1
Counselling Psychology	1
Community Services	1
Social Work	1
Nil	1
Role at organization	
Recovery Specialist	2
Community Mental Health Practitioner	2
Team Leader	2
Peer Team Leader	1
Director	1
Mental Health Support Worker	1
Years at organization	
1-3	1
3-5	3
5-8	4
8+	1
Experience in adult mental health group facilitation	
Yes	9
No	0
Previous experience of CR and CR facilitation	
Yes	0
No	9
Delivering EYM at time of interview	
Yes	0
No	9
Role in EYM	
Learning Coach	7
Manager/Supervisor	2
Status of EYM commencement and delivery by LCs *only* (during 12-month demonstration project)	
Did not commence EYM	2
Commenced and completed a full delivery of the EYM Program (Phases 1 – 4)	5

### 3.1 The EYM journey of learning

The recurring theme from LCs’ experiences of EYM training and delivery involved descriptions of it being a journey of learning and skill development (see **Figure 1**), where they naturally progressed through EYM training and implementation. The ongoing support and supervision provided to LCs also served as a strong enabler for their progression throughout both stages. The findings are presented under the relevant levels of evaluation from the Kirkpatrick Model.

**Figure 1:**
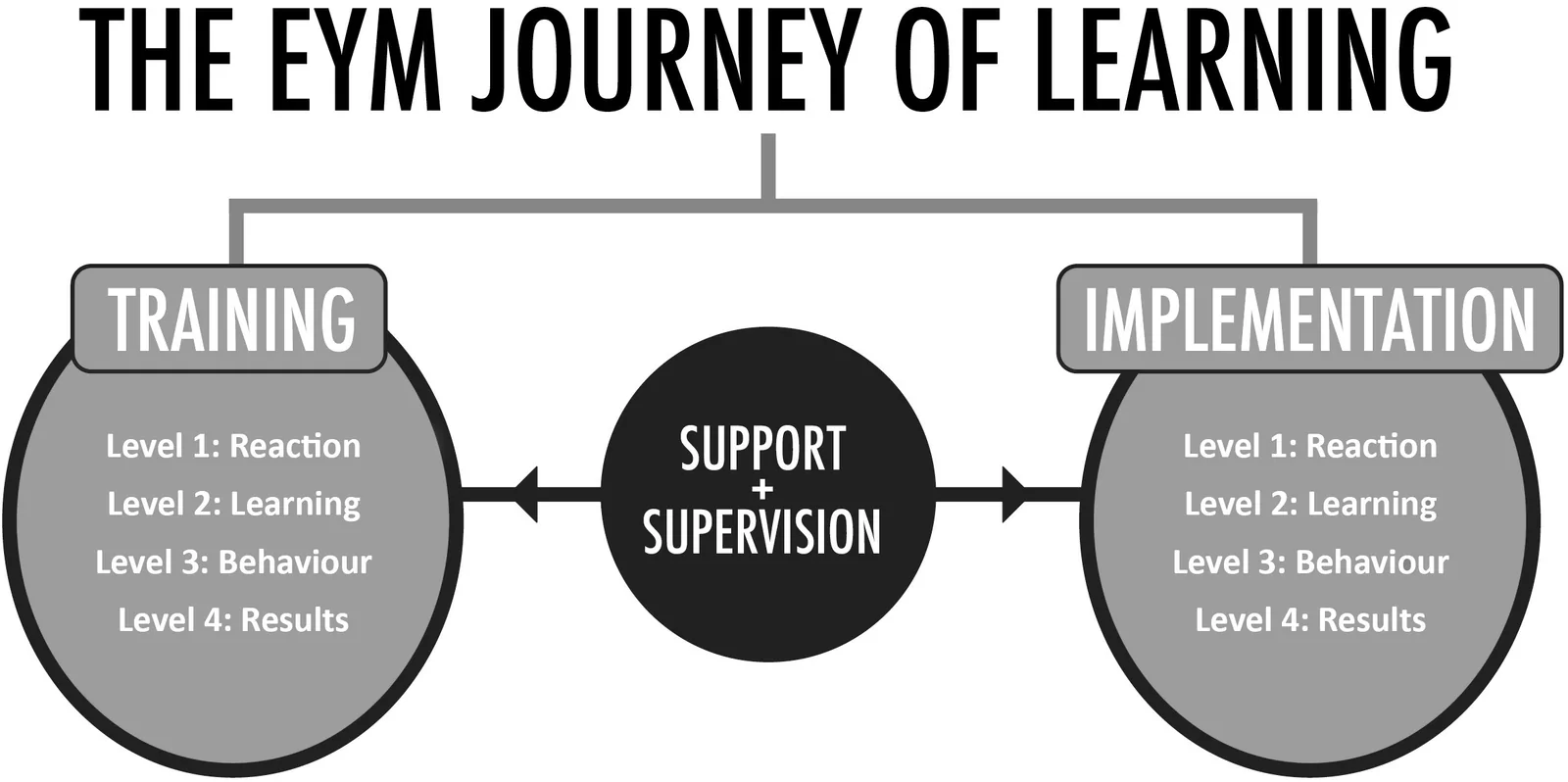
The EYM journey of learning.

#### 3.1.1 Training

**Level 1: Reaction**. After receiving the invitation to participate in training for the EYM Demonstration Project, most LCs and managers perceived the EYM program as an *‘abstract notion’* (Bel, Manager) and expressed uncertainty regarding the conceptual nature and prospective implementation of EYM into the organizations’ existing services. Stevie (LC) described that she *‘had heard a little bit about the program’* but felt she needed to research ‘*what [EYM] actually [was] and the benefits of … how that would impact our organization’.*

LCs and managers expressed positive perceptions of EYM, viewing it as an opportunity to *‘develop professionally’* (Luna, LC) and to become involved in a program that *‘was meaningful and was going to bring positive change for people’* (Lauren, LC).

All LCs and managers identified gaps in internal communication within their organization between senior leaders involved in authorizing participation in the Demonstration Project, and the line managers of participating LCs. This formed a primary barrier to having a well-informed understanding of EYM before attending the training, with Lauren (LC) expressing she felt *‘pretty clueless’* going into the training. However, the concept of EYM became *‘clearer to [LCs] until [they] got into the training itself’* (Lauren, LC).

**Level 2: Learning**. Several LCs expressed the EYM training *‘enhanced [their] existing knowledge’* (Warren, LC) and led to new learnings surrounding cognition, such as ‘*finding trends and linkages between thought patterns’* (Warren, LC), which was previously unfamiliar. Similarly, several LCs further described the EYM training enhanced their appreciation of cognition, as they explained learning about the role of *‘neuroplasticity … [and] how impactful it can be’* (Lauren, LC) within the relationship between mental health conditions and cognitive functioning.

Some LCs and managers found the EYM training encouraged reflection on their own cognitive abilities, as they *‘had to do some different tests … around our own cognition*’ (Stevie, LC), which provided insight into the ‘*reasons why we’re forgetting things … and there’s ways that you can actually adapt that to help people’* (Stevie, LC). Similarly, in training, completing certain EYM assessments as LCs themselves, provided them with first-hand insight into the EYM assessment process to be completed by EYM students, and reinforced the importance of attending to consumers’ learning and cognitive needs.

#### 3.1.2 Implementation

**Level 1: Reaction**. After the EYM training, several LCs and managers described feeling sufficiently confident to deliver Phase 1, with one LC expressing they were *‘gung-ho’* (Billy, LC) to commence implementation. Similarly, one LC described the training as engaging and *‘really relevant to the work that we do as mental health workers’* (Luna, LC). Luna further expressed the unique approach of being able to *‘deliver a learning program after being students* ***ourselves*** *and being able to learn* ***ourselves****’* as *‘satisfying’* and *‘very empowering’*.

**Level 2: Learning**. As LCs commenced implementing EYM, most described growth in their group facilitation skills. Billy (LC) expressed his initial delivery of EYM involved him *‘re-reading off the sheet a lot’*, but as EYM continued, his improved knowledge and confidence in EYM facilitation meant he *‘became a little bit smoother [and] a little bit more embedded’* with his delivery. Managers echoed this growth by noting their staff became progressively skilled in explaining *‘the whole program to clients*’ (Bel, Manager).

As service provision continued, LCs who delivered EYM described having increased knowledge of cognitive strategies to support consumers in obtaining employment and thriving in the workplace. One LC who did not deliver EYM, also reported that following the training, they developed the knowledge and skills to enable consumers to source employment.

I didn’t have much knowledge about finding a suitable job because … a number of my clients in our program have difficulties engaging with the community and living independently … so I was able to develop my skills and information to [help] my clients [find] a suitable job [post-EYM training]. (Vincent, LC)

**Level 3: Behaviour**. As LCs became more knowledgeable and skilled in delivering EYM to the consumers they support, they identified changes in their own behavior and therapeutic approaches. Before EYM, LCs described that if consumers spontaneously self-reported cognitive challenges, they would often acknowledge their experiences and provide support within their scope of practice. After the EYM training, LCs described how they could now collaboratively explore consumers’ self-reports and beliefs, to encourage consumers to recognize their capabilities to improve cognitive functioning, in areas they initially believed were unable to be targeted (e.g., memory, problem-solving).

We used to kind of approach them from a ‘Oh, okay, you can’t do it that’s all right, let me help you’…but I think what EYM really encouraged is [that] they probably do have the capacity … to do that if they tried it, or if they found a strategy to use, you know, they might be able to overcome those [challenges]. (Luna, LC)

An unexpected outcome reported by LCs included their tendency to use EYM concepts to make changes to the way they work with consumers, in addition to changes in their own lives. This was echoed by Jill (Manager), who noticed from a managerial perspective that the LCs working within the organization would discuss *‘their personal lives and … they would say … I took the EYM strategy, and I applied it … [and] the learnings were really concrete for them, so much so, that it bled into other avenues of their work and their life’*.

Most LCs described how the EYM training increased their *‘awareness’* (Billy, LC) of cognition when working with consumers, and that it enabled LCs to more thoroughly consider the reasons behind consumers’ lived experiences of cognitive challenges. LCs were then better placed to *‘coach or provide information, about why [consumers] may be feeling that way’* (Billy, LC) and propose cognitive strategies to support consumers’ cognitive function.

This, in turn, enabled LCs to hold a greater *‘positive regard’* (Lauren, LC) for consumers’ lived experiences relating to cognition and provide a more *‘holistic approach’* (Billy, LC) to mental health treatment and support.

**Implementation challenges**. For some LCs, practical and organizational challenges emerged which impacted their capacity to make behavioral changes (Level 3: Behavior) throughout EYM implementation. Some LCs and managers described managing the increased workload as challenging, as LCs’ workloads outside of EYM were *‘relatively high’* (Luna, LC).

Most LCs and managers also found the simultaneous nature of *‘learn[ing] and then teach[ing] straight away’* (Luna, LC) as challenging, which was echoed by one manager who noted this approach involved an additional *‘cognitive load’* (Jill, Manager) for her staff.

Additionally, sustaining consumer engagement throughout EYM proved challenging, due to program length, discussion of certain themes, and fluctuations in consumers’ mental health status, which occasionally affected the group learning environment. Luna (LC) described that some EYM students experienced *‘bad days and good [days]’* and *‘disengaged’* from EYM on occasions, due to the fluctuating nature of mental health conditions. However, Luna (LC) further recognized that the EYM Students she worked with had *‘persevered’* throughout the program.

**Level 4: Results**. As LCs reflected on the EYM journey, all reported positive outcomes as LCs and for consumers. The LCs and managers described consumers underwent a *‘change’* (Jill, Manager) during EYM, where they noticed a difference in their presentation, engagement and demeanor from the commencement to the completion of the program. One LC noted consumers’ *‘confidence really grew’* (Christi, LC) due to the supportive EYM environment. Jill (Manager) reported a change in one consumer who participated in the EYM program, who was initially *‘very reserved, quite shy, [and] … all of a sudden now they’re … kicking all the goals in the group, but then also started helping other people in the group’.*

Most LCs described consumers became more optimistic about employment and *‘started talking about wanting to work in an art gallery and find a volunteer [role] … [which] they hadn’t spoken about at all in their time with us in other programs’* (Jill, Manager), which differed from consumers’ attitudes pre-EYM. One LC described consumers gained hope regarding their capacities to enter/return to the workforce, as consumers began thinking *‘I can overcome this. One day I’ll find a job, and then … I’ll be able to contribute’* (Christi, LC).

Throughout EYM, some LCs and managers noted consumer withdrawal from the program; however, this was often attributed to consumers gaining employment, undertaking educational commitments or an acute change in mental state. This reflects the cyclical and dynamic nature of complex mental health conditions and the journey of recovery. For EYM Students departing from the program before completion to pursue their vocational aspirations, their partial engagement in EYM may have contributed to increased motivation or confidence to return to employment or explore educational avenues; however, this was not explored as part of this study.

We started out with six participants [EYM Students], only three made it to the end, however, even just partially doing the EYM [program], the dropout rate was only due to clients undertaking study or work commitments, so it wasn’t, it wasn’t seen as a failure. It kind of, perhaps was a bit of a catalyst for them to really decide on what they wanted to do. (Bel, Manager)

Towards the end of the Demonstration Project all LCs reported improved job satisfaction, describing the delivery of the EYM program as a *‘positive experience’* (Luna, LC). Another LC further reported that they gained *‘a lot of satisfaction out of seeing clients benefit positively from being part of the program’* (Lauren, LC).

#### 3.1.3 Support and supervision

**Level 1: Reaction.** All LCs expressed appreciation of the support and supervision provided throughout EYM training and implementation. Many LCs described it as *‘above and beyond’* (Billy, LC), with one LC describing the ‘*support that we received from WISE [IDEA] was exceptional … I think they would have to rate the highest of all the training [I have done] … I can’t fault it.* (Warren, LC).

The ongoing LC group supervision led by the EYM trainer was also perceived as *‘really helpful’* (Christi, LC) in exploring the varying EYM delivery approaches and troubleshooting implementation challenges across organizations.

**Level 2: Learning**. Several LCs mentioned WISE IDEA provided a *‘really good balance of supporting [LCs], but also’* allowing them to *‘make this [EYM] program [their] own’* (Luna, LC). This balanced approach to supervision and support during EYM, was highly beneficial and reinforced their commitment to the program throughout implementation, with the *‘hands on’* (Warren, LC) support complementing the space provided to LCs to deliver the program in their own way.

Through frequent group supervision, one LC who delivered EYM as the sole facilitator, explained this approach *‘filled that gap of not having my own LC on site*’ (Warren, LC), as he gained further insight into how other LCs navigated EYM Phases 2-4.

However, throughout implementation, some LCs mentioned wanting to more frequently review materials, to feel more *‘clear on what we’re delivering’* (Lauren, LC) and to *‘spend more time … going through … the past experiences’* (Christi, LC) of EYM implementation completed by WISE IDEA, to consolidate their own approach to EYM service delivery.

**Level 3: Behavior**. Connecting with LCs from other organizations during peer support and supervision sessions was described by some LCs as *‘encourag[ing] teamwork’* (Christ, LC) and strengthening work relationships.

Similarly, connecting with LCs from other organizations during group supervision enabled LCs to gain insight into how they delivered EYM within their organizations, and to consider alternative approaches to improve EYM implementation. Luna (LC) described that during group supervision, she recognized other LCs *‘had a lot of flexibility [in their delivery] … whereas we were very rigid’*, which prompted reflection on their current EYM facilitation approach, to better meet EYM students’ needs.

**Level 4: Results**. The EYM-specific support and supervision provided to LCs was regarded as a significant enabler in *‘bridging the gap’* (Billy, LC) of translating all EYM training into delivery. However, some LCs described encountering organizational barriers beyond their control, impeding their ability to commence EYM in their service. The implementation challenges described by LCs included limited resources, funding pressures, program length/duration, internal issues, or being the only LC trained in their service or team.

Despite the support provided by the EYM trainer to assist in navigating and problem-solving implementation challenges, two LCs faced significant barriers to translating EYM into their organization. Stevie (LC) described her organization understood the value of the EYM program, however, *‘for the reasons of our staffing, on top of the content and the length of time … [these] were some of the challenges that made it really difficult for us’* to implement the EYM program. Vincent (LC) further identified that he *‘was alone, so [he] wasn’t able to deliver the program [EYM] properly’* and was thus unable to commence delivering EYM to consumers.

## 4 Discussion

This study aimed to examine the experiences of mental health workers trained and supported to deliver EYM as part of a 12-month Demonstration Project. This is the first qualitative examination into facilitator experiences of the EYM program in Australia, which demonstrated positive outcomes at all four Kirkpatrick levels.

A significant finding of this study are the positive outcomes at Level 3 (Behavior) and Level 4 (Results) of the Kirkpatrick Model. These positive outcomes may be attributed to LCs developing an enhanced understanding of the role and significance of cognition when working with people with mental health conditions throughout the EYM training and implementation, which was further supported by managerial recounts. This, in turn, likely contributed to the LCs holding even greater positive regard and empathy for the lived experience of consumers, which ultimately encouraged the provision of more holistic mental health treatment and support. Similarly, from the LC perspective, achieving positive consumer outcomes may be associated with the development of stronger therapeutic relationships, which were formed through frequent consumer collaboration within a supportive learning environment. This finding is reflected in previous outcomes reported by Faith et al. (41) who noted positive consumer learning experiences and greater satisfaction with cognitive rehabilitation, as being achieved through strong therapeutic relationships forming in an encouraging CR environment.

An important component of the EYM program is the administration of pre and post outcome measures (29), which are returned to consumers in the form of a ‘Completion Snapshot Summary’. This summary, although not examined in this study, provides consumer-focused feedback on change that was achieved, and areas for further support. This report includes a written summary of all psychosocial, cognitive and vocational measures, which enables both the consumer and the LC to understand program outcomes and identify next steps to support their cognitive and vocational goals. Observing consumers achieve tangible and measurable outcomes, as demonstrated by the individual Completion Snapshot Summaries was found to contribute to improved job satisfaction for LCs, leading them to develop a stronger commitment to their work and place greater meaning in their roles as EYM LCs. These findings are consistent with outcomes of increased satisfaction experienced by CR therapists when delivering alternative CR programs (25) and further supports the acceptability of EYM from the LC perspective. It is important to highlight the benefits of improved job satisfaction for facilitators when undertaking training and implementation for EYM or other CR programs. This will help to promote training uptake and implementation of vocational rehabilitation programs with embedded CR in Australia and subsequently enable CR to become more widely accessible for individuals with mental health conditions. Additionally, a key contribution of this study relates to the LCs generalizing their learnings from the EYM training and its implementation to other consumers who were not directly engaged in the EYM program. While facilitator job satisfaction remains an important factor for CR training retention (25), it is important to emphasize the wider benefits of the EYM training and implementation to the mental health workforce. This study demonstrates that EYM training upskills facilitators’ capacity to address cognitive challenges for all the individuals with complex mental health conditions they work with. This adds weight to the need to expand CR and EYM training availability.

The positive outcomes of this study could be attributed to the dynamic implementation approach that utilized engaging experiential training methods which build upon the traditional didactic, resourceful and efficient approaches to CR training (18, 19). These methods were demonstrated to be more effective in engaging and sustaining LCs’ interest in training and supporting their acquisition of foundational learnings required for EYM implementation. This is consistent with the Theoretical Domains Framework (TDF) which details the influence of the person/environment interaction on the implementation of training (42). For ongoing training of EYM and other CR programs, implementing experiential training methods will continue to serve as an enabler for program acceptability and feasibility.

The challenges described by LCs and managers in this current study pertaining to limited staff capacity and increased workload are consistent with implementation research involving CR (43, 44). This study found that internal communication gaps formed an initial barrier to conveying the aims of EYM to potential LCs, leading to LCs attending the training with a limited understanding of the expectations for EYM implementation. Communicating effectively with individuals before undertaking training to promote a foundational understanding of the program and expectations would serve as an additional enabler to successful implementation and program acceptability.

Consistent with the TDF framework, this study underscores the important role of ongoing support and supervision (42) for the successful training and implementation of EYM. In this study, complementing experiential training methods with ongoing support and supervision promoted effective implementation, by providing LCs with routine access to EYM-specific support which would not otherwise be readily available within their organizations. Ongoing support needs to be a key component in CR training as it provides a social influence on behaviors and promotes self-monitoring of implementation capability (42).

Furthermore, while supervision is considered essential in upholding competent practice of CR (17), it is unclear within CR literature whether supervision for other CR programs occurs on an individual or group basis. Similarly, supervision is often viewed primarily as a key component in upholding CR fidelity, which involves ensuring facilitators can personalize CR tasks/strategies to effectively deliver CR and achieve optimal consumer outcomes (19, 24). Yet this study highlights the broader significance and benefits of the group supervision model. In this study, group supervision brought together mental health workers with varying professional backgrounds from different organizations and created a support system that alleviated the impacts of staffing shortages and increased workloads. Group supervision served to strengthen work relationships and created outlets for LCs to continually reflect on their EYM delivery and improve implementation approaches with greater efficacy and efficiency. These findings build on similar outcomes reported by Chui et al. (45) and Gardner et al. (46) who noted the benefits of peer support in group clinical supervision as mitigating the impacts of professional isolation and developing communication channels to promote competent practice. Employing *ad hoc* support alongside group supervision, as provided in this study, may also serve as a significant enabler in supporting LCs to solidify their learnings and implementation approach, thereby achieving optimal consumer outcomes. For effective supervision to take place, LCs require time to attend regular sessions, alongside support from their managers to do so. Ensuring participating organizations have capacity to allow their staff to attend supervision, remains key to maintaining EYM fidelity as the program expands. Similarly, utilizing a feasible group supervision model, such as Proctor’s (32), is necessary for the scalability of EYM as more LCs become trained to deliver the EYM program.

There are limitations within this study. Utilizing data from a small sample of mental health workers and managers who met specific inclusion criteria solely for the EYM training program and its implementation, reduces the generalizability and transferability of findings to other CR interventions. Similarly, as participants were involved in the EYM training, its implementation and they maintained ongoing supervision with the EYM Trainer, the potential for social desirability and positive response bias (47) may have influenced participant recounts and study findings. This may be particularly true for the findings surrounding their impressions (Level 1: Reaction) and the knowledge and skills gained (Level 2: Learning) from this process. Additionally, it is important to note that there was a gap of 18-months between the initial two-day EYM training and participant interviews. This retrospective recall may have affected participants’ frame of reference for the EYM training and implementation (48) and participant reports may have differed, if compared to their reflections directly after completing the Demonstration Project. Efforts were made to account for this, by designing the semi-structured interview guide to elicit LC and managerial recollections of the two-day EYM training and the most significant change experienced during this period. To more comprehensively understand the acceptability and feasibility of the EYM ‘Train-the-Coach’ model and mitigate the influence of recall bias, future studies may benefit from data triangulation of EYM fidelity measures and utilizing pre-post surveys completed by LCs, after each EYM Phase. Similarly, adopting a longitudinal approach and recruiting additional LCs and managers of LCs who were unable to implement EYM, may increase the accuracy of participant recounts and provide further examination into issues influencing the efficacy of the EYM ‘Train-the-Coach’ model. Future research should also consider examining the role of support and supervision for facilitators during the training and implementation of other CR programs, to support CR scalability in Australia.

Despite such limitations, this study displayed strengths in its sample size, which included the majority of LCs and managers within the total pool of participants involved in the EYM training and its implementation. This contributed to a range of perspectives being captured that examined the EYM ‘Train-the-Coach’ model throughout EYM training and implementation.

### 4.1 Conclusion

In summary, this study provides important insight into the enablers to successful training and implementation of the EYM program from LC and managerial perspectives and adds further value to the ongoing evaluation of the EYM program. It is important to note that study findings were derived from participants’ subjective reports of their growth and learning throughout the EYM training and implementation, rather than formal assessment or observation of their facilitation of the program. However, this study provides insight into the significance of implementing experiential training methods, and individual/group support and supervision throughout EYM training and implementation. The findings suggest this is an effective approach to enabling LCs to translate their learnings into practice and enhance their provision of holistic mental health treatment and support to consumers. Furthermore, the positive outcomes observed at Level 3 and Level 4 of the Kirkpatrick Model, further supports the feasibility and acceptability of the EYM program in community-based mental health settings.

Ultimately, the findings of this study reinforce the array of benefits of engaging in the EYM training and implementation for mental health workers, who meet the criteria required to become EYM facilitators. This includes increased knowledge and skills surrounding cognition for individuals with mental health conditions, the value of support and supervision, and LCs’ experiences of improved job satisfaction in their professional roles when observing positive consumer outcomes.

## Data Availability

The datasets presented in this article are not readily available because of identifiable information. Requests to access the datasets should be directed to Anne Miles, anne.miles@wiseemployment.com.au.
