# Supplementary material for: A qualitative exploration of mental health workers trained and supported to deliver a cognitive remediation program, Employ Your Mind (EYM)
Source: Front Psychiatry. 2026 Sep 14;17:1891467. doi: 10.3389/fpsyt.2026.1891467 (PMC13617075; doi:10.3389/fpsyt.2026.1891467)
Supplement: Supplementary file 1 [file DataSheet1.pdf]

Employ Your Mind (EYM) Demographic Questionnaire for Learning Coaches & Managers / Supervisors of EYM Learning Coaches

Please complete the following participant demographic questionnaire before the interview. Please enter or select the response that *aligns best with you*.

All information will remain private and confidential.

For any questions or further information, please contact kayla.ho@wiseemployment.com.au

1. What is your gender?

☐ Woman

☐ Man

☐ Non-binary / Gender diverse

☐ My gender identity is not listed. I identify as: ______

☐ Prefer not to say

2. What is your age (years)?

☐18 – 29 years

☐ 30 – 39 years

☐ 40 – 49 years

☐ 50 – 59 years

☐ 60 – 69 years

☐ 70 +

☐ Prefer not to say

3. What is your highest level of educational attainment?

☐ Postgraduate Degree

☐ Graduate Diploma / Graduate Certificate

☐ Bachelor's degree

☐ Advanced Diploma / Diploma

☐ Year 12 / Year 12 equivalent

☐ Year 11

☐ Year 10

☐ Year 9 or below

☐ Level of education is not identified. My highest level of educational attainment is:

☐ Prefer not to say

4. Do you have a qualification in a mental health field?

☐ Yes (see question 5)

☐ No (see question 6)

5. What is your qualification in mental health?

6. Do you have experience in adult mental health group facilitation?

☐ Yes

☐ No

7. Which organisation are you employed by?

8. How long have you been working for this organisation?

☐ 0 – 1 year

☐ 1 – 3 years

☐ 3 – 5 years

☐ 5 – 8 years

☐ 8+ years

9. What is your current role within this organisation?

10. How long have you been working in this role?

☐ 0 – 1 year

☐ 1 – 3 years

☐ 3 – 5 years

☐ 5 – 8 years

☐ 8+ years

11. What is your role within EYM under this organisation?

☐ Learning Coach (see question 12)

☐ Manager / Supervisor of Learning Coach (see question 16)

12. Are you currently delivering EYM as a Learning Coach within your organisation?

☐ Yes (see question 13)

☐ No

13. As part of the 12-month EYM demonstration project, how many Phases of EYM have you delivered?

☐ 0 – zero

☐ Phase 1

☐ Phase 2

☐ Phase 3

☐ Phase 4 (a complete EYM program)

14. Have you commenced an additional EYM program, since completing the 12-month EYM demonstration project?

☐ Yes (see question 15)

☐ No

15. How many Phases of EYM have you delivered?

☐ 0 – zero

☐ Phase 1

☐ Phase 2

☐ Phase 3

☐ Phase 4 (a complete EYM program)

16. Are you currently supervising and managing your staff deliver EYM within your organisation?

☐ Yes

☐ No

Thank you for completing this demographic questionnaire
