# Supplementary material for: A qualitative exploration of mental health workers trained and supported to deliver a cognitive remediation program, Employ Your Mind (EYM)
Source: Front Psychiatry. 2026 Sep 14;17:1891467. doi: 10.3389/fpsyt.2026.1891467 (PMC13617075; doi:10.3389/fpsyt.2026.1891467)
Supplement: Supplementary file 2 [file DataSheet2.pdf]

Participant Interview Guide:

Introduction

Hi, my name is Kayla, and I am a 4th year Occupational Therapy student at Monash University. As part of my Research Honours project, titled *"Exploring the Experiences of Mental Health Workers Trained and Supported to Deliver a Cognitive Remediation Program, Employ Your Mind (EYM)"*, I am conducting an in-depth semi-structured interview with Learning Coaches and managers involved from your organisation. I appreciate the time you have taken to participate in this interview and providing informed consent.

The purpose of this interview is to explore your perceptions and experiences on areas such as:

- Learning Coach perceptions of the EYM training;
- Learning Coach perceptions of EYM service delivery and;
- Manager perceptions of EYM training of staff and their service delivery.

A participant interview guide would have been emailed to yourself prior to the interview, that described the types of questions you will be asked. The current interview is expected to take 1 hour to a maximum of 1.5 hours.

The findings of the interview will inform the ongoing evaluation of EYM delivered by WISE IDEA, and how the program contributes to change and improvements within the mental health workforce, and mental health workers' approach to working with individuals with severe mental illness in vocational settings.

Your identity will remain protected, and you will be asked to select a pseudonym of preference, for future reporting of interview data. As outlined in the explanatory statement and informed consent form, the interview will be recorded and transcribed. All audio recordings, responses, and transcriptions will remain confidential and anonymous.

Only applicable sets of questions within each topic will be asked, depending on the demographic information you have provided. I will alert you when the final question of the interview has been reached, before asking for your response. I seek to explore your experiences and perceptions within such topics, and I encourage you to provide honest answers.

If you do not understand a question at any point, feel free to pause and I will clarify and explain the question. Throughout the interview, I may ask follow-up questions to understand your experience further.

Finally, if you do not wish to answer a question or continue with the interview at any point in time, please let me know and I will terminate the interview and audio recording immediately.

Do you have any questions? *Participant answers YES or NO*

(Proceed to ask questions from within the relevant Part/s, according to the participant)

Part 1:

Research Question - What are the mental health workers' perceptions of the EYM training?

***Questions dedicated for ALL Learning Coaches who undertook the EYM Training**

1. Could you please describe how you became aware of the EYM training?
    a. Were there any expectations going into the training?
    b. Did the EYM training meet your expectations?

2. From your point of view, what support did you receive from WISE IDEA throughout EYM training?
    a. In your opinion, what was the most significant form of support?
    b. Why was this most significant to you?
    c. Is there anything additional that WISE IDEA could provide that would support your training of EYM?

3. Have you experienced any changes in your knowledge, skills, and/or attitudes, since undertaking the EYM training?
   a. Across these changes you have described, what do you think was the most significant change of all?
   b. Were there any other changes you experienced within yourself, when undertaking EYM training?
   c. Was there anything that helped you undertake the EYM training?
   d. Did you experience any barriers when undertaking EYM training?

Part 2:

Research Question - What are the mental health workers' perceptions of how delivering EYM has changed how they work with service users?

****Questions dedicated for Learning Coaches who <u>have delivered</u> EYM in their service**

1. Looking over your delivery of EYM, what do you think was the most significant change within yourself as a learning coach?
   a. Why was this change most significant?
   b. What caused this change?
   c. What other areas of change did you notice within yourself as a learning coach, having delivered EYM?

2. Were there any changes you experienced in your professional role, when delivering EYM?
   a. Across these changes, what was the most significant change you experienced in your professional role, when delivering EYM?
   b. How did this significant change affect your approach to working with EYM service users?

c. Did delivering EYM affect your job satisfaction?

3. Were you satisfied with the support provided by WISE IDEA throughout your delivery of EYM?

   a. Is there anything additional that WISE IDEA could provide that would support your delivery of EYM?

Part 3:

Research Question - What are managers' perceptions of service delivery following the EYM training of their mental health workers?

Part 3a: ***Questions dedicated for managers whose staff undertook the EYM Training and <u>have delivered</u> EYM within their service

1. How many staff undertook the EYM-training under your organisation?
   a. Were you involved in setting up the EYM training for your staff?
   b. Why did you think this training would be important for your staff?

2. Could you please describe your role as a manager during the EYM training of your staff?

3. Could you please describe your role as a manager throughout the service delivery of EYM?

4. Have you noticed any changes in the knowledge, skills, and/or attitudes of your staff since they undertook the EYM training?

   a. Across these changes you have described, what do you think was the most significant change of all?
   b. Why was this change most significant?

5. Over the course of EYM delivery, were there any changes you noticed in your staff throughout their <u>delivery</u> of EYM?
   a. What was the most significant change within your staff throughout service delivery?
   b. Why was this change most significant?
   c. Has this change within your staff sustained overtime?

6. What other forms of change did you notice during service delivery of EYM?
   a. Was there anything that supported the delivery of EYM within your service?
   b. Were there any barriers throughout the delivery of EYM within your service?

Part 3b: ****Questions dedicated for managers whose staff undertook the EYM Training but <u>have NOT delivered</u> EYM within their service

1. How many staff undertook the EYM-training under your organisation?
   a. Were you involved in setting up the EYM training for your staff?
   b. Why did you think this training would be important for your staff?
   c. Did you have role in supporting your staff during the EYM training?
2. Did you notice any changes in the knowledge, skills and/or attitudes of your staff who undertook the EYM training?
   a. Across these changes, what was the most significant change overall?
   b. Why was this change most significant?

3. What is your perception of why your staff who undertook the EYM training, did not deliver EYM within your service?
    a. Do you think there is anything that could have been done differently, that would have enabled your staff to deliver EYM?

Closing Statement

[Alert participant that we have arrived on the final question of the interview]

Thank you for your valued time in participating in this interview. Do you have any questions about the interview process and next steps?

[Pause for response]

I am going to be stopping the recording now, and you are welcome to contact me or the research team via the details I have provided, for any further questions.
